# Sexual Risk Behavior Differences Among Sexual Minority High School Students — United States, 2015 and 2017

**DOI:** 10.15585/mmwr.mm6736a3

**Published:** 2018-09-14

**Authors:** Catherine N. Rasberry, Richard Lowry, Michelle Johns, Leah Robin, Richard Dunville, Sanjana Pampati, Patricia J. Dittus, Alexandra Balaji

**Affiliations:** ^1^Division of Adolescent and School Health, National Center for HIV/AIDS, Viral Hepatitis, STD, and TB Prevention, CDC; ^2^Oak Ridge Institute for Science and Education (ORISE), Oak Ridge, Tennessee; ^3^Division of STD Prevention; National Center for HIV/AIDS, Viral Hepatitis, STD, and TB Prevention, CDC; ^4^Division of HIV/AIDS Prevention, National Center for HIV/AIDS, Viral Hepatitis, STD, and TB Prevention, CDC.

Sexual minority youths (i.e., those identifying as gay, lesbian, bisexual, or another nonheterosexual identity or reporting same-sex attraction or sexual partners) are at higher risk than youths who are not sexual minority youth (nonsexual minority youth) for negative health behaviors and outcomes, including human immunodeficiency virus (HIV) infection, other sexually transmitted diseases (STDs), pregnancy (*1*),* and related sexual risk behaviors (*2*). Less is known about sexual risk behavior differences between sexual minority youth subgroups. This is the first analysis of subgroup differences among sexual minority youths using nationally representative Youth Risk Behavior Survey (YRBS) data. CDC analyzed pooled data from the 2015 and 2017 cycles of the national YRBS, a cross-sectional, school-based survey assessing health behaviors among U.S. students in grades 9**–**12. Analyses examined differences in eight sexual risk behaviors between subgroups of sexual minority youths and nonsexual minority youths, as well as within sexual minority youths. Logistic regression models controlling for race/ethnicity and grade found that bisexual females and “not sure” males reported higher prevalences for many behaviors than did heterosexual students. For behavior-based subgroups, the largest number of differences were seen between students who had sexual contact with both sexes compared with students with only opposite-sex sexual contact. Findings highlight subgroup differences within sexual minority youths that could inform interventions to promote healthy behavior.

The national YRBS is a biennial, school-based survey of U.S. high school students. To achieve a sufficient sample size for sexual minority youth subgroup analysis, 2015 and 2017 national YRBS data were pooled. For each year, a nationally representative sample of students in grades 9–12 attending public and private schools was selected using a three-stage cluster sample design (*3*). In 2015, overall response rate and sample size were 60% and 15,624, respectively; in 2017, overall response rate and sample size were 60% and 14,765, respectively. Data were weighted to yield nationally representative estimates. The combined sample included 30,389 survey responses. Students completed the self-administered questionnaire during one class period, recording responses onto computer-scannable booklets/answer sheets. Survey procedures protected students’ privacy through anonymous and voluntary participation. Local parental permission procedures were followed.

Sexual minority youths were defined by self-reported sexual identity and behavioral characteristics. Sexual identity was assessed by the question, “Which of the following best describes you?” (response options: heterosexual; gay or lesbian; bisexual; not sure). Sex of sexual contacts was assessed through two questions: “During your life, with whom have you had sexual contact?” (response options: I have never had sexual contact; females; males; females and males) and “What is your sex?” (response options: female; male). A 3-level categorical variable was created to describe sex of sexual contacts (same-sex only; both sexes; opposite-sex only). Eight questions assessed sexual risk behaviors. Multiple-choice responses were dichotomized to create behavioral measures: ever had sexual intercourse, had first sexual intercourse before age 13 years (early sexual debut), had sexual intercourse with four or more persons during their life (≥4 sex partners), had sexual intercourse during the 3 months preceding the survey (currently sexually active), did not use a condom during last sexual intercourse (no condom use), did not use any method to prevent pregnancy during last sexual intercourse (no pregnancy prevention method use), drank alcohol or used drugs before last sexual intercourse (alcohol/drug use before sex), and never been tested for HIV.

Analyses used statistical software to account for the complex sampling design. Unadjusted prevalence estimates with 95% confidence intervals (CIs) were calculated using Taylor series linearization. Sex-stratified logistic regression models produced adjusted prevalence ratios (APRs) comparing each sexual minority youth subgroup with heterosexual students or students with only opposite-sex sexual contact. Models controlled for race/ethnicity and grade, and except for the model predicting ever had sexual intercourse, were limited to students who had ever had sexual intercourse. Differences in risk behavior prevalence between sexual minority youth subgroups were tested using linear contrast t-tests. Statistical tests were considered significant if p<0.05 or 95% CIs did not include 1.0.

Across identity-based subgroups, unadjusted prevalence estimates for having ever had sexual intercourse ranged from 26.9% to 51.4% for females and from 33.9% to 47.8% for males (Table 1). Identity-based sexual minority youth subgroups were more likely than were heterosexual students to engage in sexual risk behaviors (Table 2). Bisexual females were more likely than were heterosexual females to report having had sexual intercourse (APR = 1.41), early sexual debut (APR = 2.43), ≥4 sex partners (APR = 1.69), no condom use (APR = 1.17), no pregnancy prevention method use (APR = 1.49), and alcohol/drug use before sex (APR = 1.36). Males who were not sure about their sexual identity were more likely than were heterosexual males to report early sexual debut (APR = 2.33), ≥4 sex partners (APR = 1.47), no pregnancy prevention method use (APR = 2.03), and alcohol/drug use before sex (APR = 1.73). Lesbian or bisexual females were more likely than were females who were not sure about their sexual identity to report having had sexual intercourse, no condom use, and no pregnancy prevention method use. Gay or bisexual males were more likely than were males who were not sure to report having had sexual intercourse and not using pregnancy prevention. Gay/lesbian students were more likely than were bisexual students to report not using pregnancy prevention, and among females, not using condoms.

**TABLE 1:** Unadjusted weighted prevalence of sexual risk behaviors among sexual minority youths by identity- and behavior-based subgroups—National Youth Risk Behavior Survey, United States, 2015–2017

Sexual risk behavior	Sexual identity % (95% CI)				Sex of sexual contacts % (95% CI)		
	Gay or lesbian	Bisexual	Not sure	Heterosexual	Same sex only	Both sexes	Opposite sex only
**Females**							
Ever had sexual intercourse	48.4 (41.5–55.4)	51.4 (47.0–55.8)	26.9 (22.3–32.0)	37.3 (34.4–40.2)	66.2 (57.8–73.7)	72.6 (67.4–77.3)	77.7 (74.9–80.3)
Had first sexual intercourse before age 13 years*	11.1 (5.0–23.1)	11.2 (8.6–14.5)	12.0 (7.4–18.9)	3.8 (3.1–4.8)	15.2 (8.1–26.7)	13.2 (10.5–16.4)	3.7 (3.0–4.6)
Had sexual intercourse with ≥4 persons*^,†^	25.2 (15.6–37.9)	30.7 (26.5–35.2)	29.4 (18.7–43.0)	19.5 (18.0–21.1)	19.6 (11.9–30.6)	43.9 (38.0–50.0)	18.1 (16.5–19.8)
Currently sexually active*^,§^	69.3 (56.5–79.6)	71.4 (66.7–75.7)	68.4 (58.1–77.1)	77.7 (75.9–79.4)	65.7 (54.8–75.2)	77.5 (73.3–81.2)	76.3 (74.6–78.0)
Did not use condom during last sexual intercourse*^,¶^	82.5 (68.6–91.0)	51.7 (46.6–56.7)	42.5 (31.3–54.5)	44.6 (42.3–47.0)	—	56.5 (51.9–61.1)	43.6 (41.4–45.9)
Did not use any method to prevent pregnancy during last sexual intercourse*^,^**	68.5 (56.2–78.7)	21.3 (17.5–25.6)	15.4 (9.8–23.5)	13.3 (11.5–15.4)	—	21.4 (18.0–25.3)	13.3 (11.5–15.4)
Drank alcohol or used drugs before last sexual intercourse*	18.5 (9.6–32.4)	20.4 (17.4–23.9)	31.2 (21.1–43.4)	15.1 (13.6–16.7)	17.8 (10.0–29.6)	28.2 (23.9–32.9)	14.3 (12.9–15.9)
Never been tested for HIV*^,††^	80.3 (68.8–88.3)	73.3 (68.5–77.5)	84.1 (76.8–89.5)	79.8 (77.6–81.7)	77.6 (65.9–86.1)	69.1 (64.4–73.4)	80.5 (78.4–82.4)
**Males**							
Ever had sexual intercourse	47.8 (39.3–56.3)	42.7 (35.3–50.5)	33.9 (27.5–41.0)	42.5 (40.0–44.9)	83.3 (75.3–89.1)	77.3 (68.9–84.0)	78.9 (76.3–81.2)
Had first sexual intercourse before age 13 years*	21.4 (11.3–36.8)	18.1 (10.4–29.6)	29.7 (20.6–40.6)	11.3 (9.9–13.0)	26.1 (15.1–41.1)	28.1 (19.6–38.6)	11.1 (9.6–12.7)
Had sexual intercourse with ≥4 persons*^,†^	28.8 (20.2–39.2)	28.3 (19.0–39.8)	46.1 (33.1–59.7)	30.3 (27.9–32.8)	25.0 (17.3–34.7)	43.0 (33.6–52.9)	30.2 (27.9–32.7)
Currently sexually active*^,§^	67.1 (56.1–76.5)	60.6 (49.5–70.7)	72.4 (61.6–81.1)	70.4 (68.5–72.3)	66.2 (54.1–76.4)	66.1 (56.1–74.8)	70.5 (68.5–72.3)
Did not use condom during last sexual intercourse*^,¶^	54.4 (37.8–70.0)	43.7 (34.3–53.6)	45.1 (32.6–58.1)	35.1 (32.9–37.3)	58.4 (46.0–69.8)	46.1 (35.9–56.6)	35.1 (32.9–37.4)
Did not use any method to prevent pregnancy during last sexual intercourse*^,^**	48.8 (33.0–64.7)	18.3 (11.3–28.4)	25.1 (14.9–39.0)	10.8 (9.6–12.2)	—	21.6 (14.6–30.6)	10.8 (9.4–12.4)
Drank alcohol or used drugs before last sexual intercourse*	15.8 (8.4–27.9)	19.9 (11.9–31.3)	36.5 (25.2–49.5)	20.5 (18.7–22.4)	13.5 (7.1–24.2)	30.3 (22.2–39.9)	20.6 (18.8–22.5)
Never been tested for HIV*^,††^	69.7 (53.9–81.9)	84.4 (74.6–90.9)	82.1 (71.6–89.2)	87.0 (85.2–88.7)	78.0 (63.9–87.7)	82.4 (75.8–87.5)	87.0 (85.1–88.8)

**Abbreviations:** AIDS = acquired immunodeficiency virus; CI = confidence interval; HIV = human immunodeficiency virus.

*Analysis conducted among only students who ever had sexual intercourse. Analyses were not conducted for no condom use among female students with only same-sex sexual contact or for no pregnancy prevention use among male or female students with only same-sex sexual contact. In addition, students who had no sexual contact are excluded from the analyses by sex of sexual contact.

^†^ During their life.

^§^ Had sexual intercourse with at least one person during the 3 months before the survey.

^¶^ Question asked about condom use by “you or your partner.”

** Question asked about method used for pregnancy prevention by “you or your partner.”

^††^ Question asked, “Have you ever been tested for HIV, the virus that causes AIDS? (Do not count tests done if you donated blood.)”

**TABLE 2:** Adjusted prevalence ratios* for sexual risk behaviors among sexual minority youths by identity- and behavior-based subgroups (using heterosexual students and students with only opposite-sex partners as the referents)—National Youth Risk Behavior Survey, United States, 2015–2017

Sexual risk behavior	Sexual identity APR (95% CI)			Sex of sexual contacts APR (95% CI)	
	Gay or lesbian	Bisexual	Not sure	Same sex only	Both sexes
**Females**					
Ever had sexual intercourse	1.26^§,¶^ (1.09–1.47)	1.41^§,¶^ (1.29–1.55)	0.75^§^ (0.64–0.89)	0.86^††^ (0.75–0.98)	0.95 (0.90–1.01)
Had first sexual intercourse before age 13 years^†^	2.44^§^ (1.06–5.61)	2.43^§^ (1.66–3.57)	2.68^§^ (1.61–4.46)	2.97^††^ (1.42–6.19)	3.05^††^ (2.20–4.24)
Had sexual intercourse with ≥4 persons^†^	1.39 (0.89–2.16)	1.69^§^ (1.43–2.00)	1.62^§^ (1.08–2.43)	1.11^¶¶^ (0.65–1.92)	2.49^††^ (2.10–2.96)
Currently sexually active^†^	0.92 (0.77–1.08)	0.94^§^ (0.88–1.00)	0.90 (0.78–1.03)	0.89 (0.76–1.05)	1.03 (0.98–1.08)
Did not use condom during last sexual intercourse^†^	1.85^§,¶,^** (1.58–2.16)	1.17^§^ (1.05–1.30)	0.96 (0.72–1.30)	—	1.30^††^ (1.18–1.43)
Did not use any method to prevent pregnancy during last sexual intercourse^†^	4.88^§,¶,^** (3.77–6.32)	1.49^§^ (1.20–1.85)	1.07 (0.68–1.69)	—	1.52^††^ (1.27–1.81)
Drank alcohol or used drugs before last sexual intercourse^†^	1.27 (0.70–2.32)	1.36^§^ (1.13–1.63)	2.13^§^ (1.45–3.13)	1.09^¶¶^ (0.60–1.99)	1.94^††^ (1.58–2.38)
Never been tested for HIV^†^	1.03 (0.92–1.15)	0.92^§,¶^ (0.87–0.98)	1.06 (0.98–1.14)	0.98 (0.87–1.10)	0.86^††^ (0.81–0.91)
**Males**					
Ever had sexual intercourse	1.11^¶^ (0.92–1.33)	1.02 (0.84–1.23)	0.78^§^ (0.63–0.96)	1.05 (0.97–1.15)	0.99 (0.90–1.09)
Had first sexual intercourse before age 13 years^†^	1.86 (1.00–3.48)	1.58 (0.92–2.72)	2.33^§^ (1.55–3.51)	1.96^††^ (1.10–3.50)	2.64^††^ (1.86–3.75)
Had sexual intercourse with ≥4 persons^†^	0.93 (0.63–1.36)	1.00 (0.68–1.47)	1.47^§^ (1.06–2.04)	0.76^¶¶^ (0.53–1.10)	1.48^††^ (1.19–1.85)
Currently sexually active^†^	0.94 (0.80–1.09)	0.86 (0.71–1.03)	1.02 (0.88–1.18)	0.98 (0.84–1.15)	0.94 (0.81–1.08)
Did not use condom during last sexual intercourse^†^	1.54^§^ (1.13–2.11)	1.24 (0.99–1.56)	1.24 (0.90–1.72)	1.67^††^ (1.36–2.07)	1.34^††^ (1.02–1.77)
Did not use any method to prevent pregnancy during last sexual intercourse^†^	4.38^§,¶,^** (3.11–6.17)	1.72^§^ (1.07–2.76)	2.03^§^ (1.18–3.49)	—	2.12^††^ (1.45–3.11)
Drank alcohol or used drugs before last sexual intercourse^†^	0.73^¶^ (0.39–1.38)	0.94^¶^ (0.57–1.53)	1.73^§^ (1.15–2.61)	0.52^††,¶¶^ (0.28–0.99)	1.45^††^ (1.02–2.05)
Never been tested for HIV^†^	0.81^§^ (0.67–0.98)	0.96 (0.87–1.06)	0.92 (0.81–1.04)	0.89 (0.76–1.04)	0.93^††^ (0.86–1.00)

**Abbreviations:** APR = adjusted prevalence ratio; CI = confidence interval; HIV = human immunodeficiency virus.

*Logistic regression adjusted for race/ethnicity and grade. Statistical significance is indicated when p<0.05 or 95% CI does not include 1.0. Analyses were not conducted for no condom use among females with only same-sex sexual contact or for no pregnancy prevention method use among male or female students with only same-sex sexual contact.

^†^ Among students who ever had sexual intercourse.

^§^ Linear contrast t-tests reveal this sexual minority youth subgroup is significantly different from heterosexual students.

^¶^ Linear contrast t-tests reveal this sexual minority youth subgroup is significantly different from students who are not sure of their sexual identity.

** Linear contrast t-tests reveal this sexual minority youth subgroup is significantly different from bisexual students.

^††^ Linear contrast t-tests reveal this sexual minority youth subgroup is significantly different from students who had sexual contact with the opposite sex only.

^¶¶^ Linear contrast t-tests reveal this sexual minority youth subgroup is significantly different from students who had sexual contact with both sexes.

Across behavior-based subgroups, unadjusted prevalence estimates for having ever had sexual intercourse ranged from 66.2% to 77.7% for females and from 77.3% to 83.3% for males (Table 1). Students who had sexual contact with both sexes were more likely than were those with only opposite-sex sexual contact to report early sexual debut (females, APR = 3.05; males, APR = 2.64), ≥4 sex partners (females, APR = 2.49; males APR = 1.48), no condom use (females, APR = 1.30; males, APR = 1.34), no pregnancy prevention method use (females, APR = 1.52; males, APR = 2.12), and alcohol/drug use before sex (females, APR = 1.94; males, APR = 1.45); these students were more likely than were students with only same-sex sexual contact to report ≥4 sex partners and alcohol/drug use before sex (Table 2). Students with only same-sex sexual contact were more likely than were students with only opposite-sex sexual contact to report early sexual debut (females, APR = 2.97; males, APR = 1.96), and among males, no condom use (APR = 1.67).

## Discussion

Consistent with 2017 YRBS data showing higher prevalence of sexual risk behaviors among sexual minority youths than among nonsexual minority youths (*2*), this analysis of pooled 2015 and 2017 YRBS data found that identity- and behavior-based sexual minority youth subgroups were more likely than were their nonsexual minority counterparts to report a range of sexual risk behaviors. Among identity-based groups, bisexual females and males who were not sure more frequently reported higher prevalences of risk behaviors than did heterosexual students, indicating students not identifying as gay/lesbian or heterosexual might exhibit higher levels of sexual risk behavior. For bisexual females, this aligns with previous research (*4*); however, interpretations of findings for males who reported that they were not sure of their sexual identity can be less clear. “Not sure” might reflect a respondent’s uncertainty about his/her own sexual identity or uncertainty about the meaning of the question or response options. Findings reveal differences between “not sure” and heterosexual peers, as previously documented (*2*), but also between “not sure” and both gay/lesbian and bisexual subgroups for several behaviors. Higher risk behavior prevalence in sexual minority youths reporting sexual contact with both sexes compared with students with only opposite-sex sexual contact was consistent with previously reported findings (*5*,*6*).

The prevalences of no pregnancy prevention method use and, for females, no condom use were higher among gay/lesbian than among bisexual students. Lesbian females with only same-sex sexual contact might perceive reduced need for condoms to prevent pregnancy. However, researchers have documented both increased pregnancy risk among lesbians (*1*) and identity-behavior discordance among adolescents, indicating that sexual identity might not reflect behavior (*7*).

Risk behavior differences based on behavior-based subgroups revealed that females and males who had sexual contact with both sexes were at higher risk than were their peers with only same-sex sexual contact for having had ≥4 sex partners and alcohol/drug use before sex. These findings align with previously published studies documenting that students who had sexual contact with both sexes were at higher risk than were students with only same-sex sexual contact for multiple sexual risk behaviors (*5*,*6*). In addition, higher risk of no condom use among males with only same-sex sexual contact compared with those with only opposite-sex sexual contact is particularly concerning because of the higher HIV/STD risk among this group.^†^

The findings in this report are subject to at least three limitations. First, these findings represent students in public or private schools and not all youths of similar ages, including those not in school. Nationwide, in 2013, approximately 5% of persons aged 16–17 years were not enrolled in high school and lacked a high school credential^§^; however, sexual minority youths might represent a disproportionate percentage of high school dropouts and other youths absent from or not attending school (*8*). Second, although questions exhibited good reliability in a test-retest study (*9*), over- or underreporting of behaviors cannot be estimated. Finally, because sexual contact was not defined, students might have considered various sexual activities when responding to this question, including involuntary sexual contact.

These findings support the importance of considering both sexual identity and behavior in assessment of risk among sexual minority youths, as other researchers have encouraged (*7*,*10*). Sexual minority youth subgroups report differential risk levels within sexual minority youths and between sexual minority youths and nonsexual minority youths. Understanding these differences within sexual minority youths might help public health practitioners tailor sexual risk reduction and health promotion interventions to meet subgroup prevention needs by aligning intervention focus to the specific profiles of each subgroup and ensuring content is relevant to their experiences and needs.

**Summary**

**What is already known about this topic?**

Sexual minority youths are at higher risk than are nonsexual minority youths for human immunodeficiency virus infection, sexually transmitted diseases, pregnancy, and related risk behaviors. Less is known about risk differences among sexual minority youth subgroups.

**What is added by this report?**

Among sexual minority youths, risk behaviors were more prevalent among bisexual females and males who were not sure than among their heterosexual peers as well as among students who had sexual contact with both sexes than among those with only same-sex sexual contact.

**What are the implications for public health practice?**

Better understanding differential risk within sexual minority youths might help public health practitioners tailor sexual risk reduction interventions for sexual minority youths.

## Footnotes

* https://www.cdc.gov/healthyyouth/disparities/smy.htm.

† https://www.cdc.gov/hiv/group/age/youth/index.html.

§
https://nces.ed.gov/pubs2016/2016117rev.pdf .
